# Emerging Biomarkers for Immunotherapy in Glioblastoma

**DOI:** 10.3390/cancers14081940

**Published:** 2022-04-12

**Authors:** Nadia Mensali, Else Marit Inderberg

**Affiliations:** Translational Research Unit, Department of Cellular Therapy, Oslo University Hospital, 0379 Oslo, Norway; nadia.mensali@rr-research.no

**Keywords:** glioblastoma, immunotherapy, biomarkers, tumour infiltrating lymphocytes, immunoprofiling

## Abstract

**Simple Summary:**

Immunotherapy has shown clinical benefits in several solid cancers; still, glioblastoma remains very challenging to treat. Glioblastoma is the most frequent brain cancer and displays great heterogeneity. The standard of care has remained the same for over fifteen years, and to overcome the therapeutic limitations, emerging immune correlates of therapy responses and improved prognosis should be further developed for a more personalized therapy approach and increased clinical responses.

**Abstract:**

Immunotherapy has shown clinical benefits in several solid malignancies—in particular, melanoma and non-small cell lung cancer. However, in other solid tumours such as glioblastoma (GBM), the response to immunotherapy has been more variable, and except for anti-PD-1 for patients with microsatellite instable (MSI)+ cancers, no immunotherapy is currently approved for GBM patients. GBM is the most common and most aggressive brain cancer with a very poor prognosis and a median overall survival of 15 months. A few prognostic biomarkers have been identified and are used to some extent, but apart from MSI, no biomarkers are used for patient stratification for treatments other than the standard of care, which was established 15 years ago. Around 25% of new treatments investigated in GBM are immunotherapies. Recent studies indicate that the use of integrated and validated immune correlates predicting the response and guiding treatments could improve the efficacy of immunotherapy in GBM. In this review, we will give an overview of the current status of immunotherapy and biomarkers in use in GBM with the main challenges of treatment in this disease. We will also discuss emerging biomarkers that could be used in future immunotherapy strategies for patient stratification and potentially improved treatment efficacy.

## 1. Introduction

Glioblastoma multiforme (GBM) is the most prevalent primary brain tumour of the central nervous system (CNS) in adults. The standard of care (SOC) in newly diagnosed GBM is a treatment regimen that consists of tumour surgical resection followed by concurrent radiotherapy (RT) and systemic temozolomide (TMZ) chemotherapy. Despite this aggressive multimodal treatment, the outcomes are still unsatisfactory, and the prognosis for GBM patients remains dismal, with 7–8 months of median progression-free survival (PFS), a median overall survival (OS) below 15 months, and a 5-year survival rate of only 6.8% [1]. Virtually, all GBMs will relapse; the tumour progresses aggressively and rapidly, and no standard of care for relapsed or recurrent GBM has been established yet. GBMs invariably recur and become resistant to current therapeutic regimens, representing one of the major barriers to the effectiveness of treatments. The failure of conventional therapies in the management of brain tumours highlights the urgent need for novel therapeutic options and interventions. In this scenario, the success of cancer immunotherapy in other aggressive, advanced solid tumours, such as melanoma and non-small cell lung cancer, has raised great hope for GBM and encouraged the investigation of experimental immunotherapeutic approaches. Different immunotherapeutic modalities, including checkpoint inhibitors, vaccines, adoptive T-cell transfer, chimeric antigen receptor (CAR) T-cell therapy, and oncolytic virus, have been combined with conventional therapies to enhance the clinical benefits of the standard of care and have been interrogated as potential novel therapeutic options for GBM [2]. Currently, around 25% of the ongoing clinical trials in GBM are exploiting immunotherapeutic interventions. Despite the promising results achieved in preclinical studies, numerous clinical trials have failed to reach their clinical endpoints [3], and to date, no immunotherapy has been approved as a first-line treatment in GBM; indeed, the majority of GBM patients have been shown to be resistant to immunotherapies [4,5,6]. There is no doubt that immunotherapeutic approaches can induce immunological anti-tumour responses, as observed in several GBM patients; unfortunately, these responses are, in most cases, limited. Nevertheless, there is a small fraction of GBM patients who experience more durable responses and are still alive 2 years after diagnosis, and in fewer cases, they survive even longer [7,8,9]. GBM long-term survivors constitute proof that GBM patients might benefit from immunotherapy. Deciphering the immunological and tumour characteristics of long-term survival patients and understanding the differences with non-responder patients will provide precious information for guiding the optimization of treatments, selection of eligible patients and in general, improve the clinical management of GBM.

In this review, we will provide a brief introduction to the unique features of GBM and their implications in the major mechanisms of immunotherapy resistance. We will review the current immunotherapeutic strategies by highlighting the most relevant clinical studies and their immunological correlates. We will discuss the remaining challenges of GBM immunotherapy and novel immunotherapeutic approaches designed to overcome the current obstacles to effective immunotherapy against GBM. We will also give an overview of the current biomarkers in use in the management of glioblastoma, explore emerging immune biomarkers and discuss their potential roles in guiding patient stratification, as well as clinical study planning. Understanding biomarkers for appropriate patient selection, as well as tumour progression, is necessary for the implementation of immunotherapy for GBM.

## 2. Current Prognostic Biomarkers in Clinical Use

The 2016 World Health Organization (WHO) Classification of CNS tumours formally incorporated molecular characteristics into the definitions of many of these tumours, including GBM [10]. Of the very few biomarkers that are used clinically in GBM today, the DNA repair enzyme O(6)-methylguanine-DNA methyltransferase (MGMT) promoter methylation predicting response to chemotherapy is the one that is the most implemented but is not used very actively, as there is a lack of treatment options. For example, while an MGMT promoter unmethylated isocitrate dehydrogenase (IDH) wild type (WT) GBM patient is likely to succumb to the disease within a year, patients with an IDH-mutated, highly MGMT promoter methylated tumour have a fair chance at living beyond 5 years [11]. However, these estimates are very uncertain. Improved clarification of the expected prognosis is exceptionally important in patient care. IDH mutations are considered a marker of good prognosis in GBM, but the association with improved prognosis is weak, and mutations occur in less than 10% of patients and mainly in the younger ones [12,13]. The majority of IDH mutations are seen in secondary versus primary GBM, and IDH-mutated patients frequently also have MGMT promoter methylation and respond better to chemotherapy [14]. Epidermal growth factor receptor (EGFR) is the most common genetic alteration in primary GBM, with around two-thirds of patients expressing a constitutively activated mutant form, EGFRvIII [15]. The EGFRvIII mutation has been targeted by small molecule inhibitors, vaccines and CAR-T therapy but can be easily lost by the tumour, and a clear prognostic or predictive value has not been demonstrated [16,17]. Around 75% of primary GBM have promoter mutations in the human telomerase reverse transcriptase gene (hTERT) which have been found to negatively influence survival in MGMT unmethylated patients [18]. In summary, unmethylated MGMT promoter and IDH WT markers define the largest group of patients with the worse prognostic outcome.

Overall, clinical experience supports the value of the available markers. Even though they are not currently used extensively in patient stratification, combined with other biomarkers, they may be indicative of personalized treatments and open up access of potential responsive patients to novel therapies under evaluation, as exemplified in melanoma [19]. The discovery of immune-linked biomarkers that predict what other treatments could be combined with the standard treatments to further improve their efficacy is thus necessary [20,21,22]. This could, for example, be immune checkpoint blockade (ICB), which has so far been disappointing in GBM apart from in a few subgroups of patients [23].

## 3. Immunotherapy for Glioblastoma

Recently, immunotherapy has emerged as the standard modality of cancer care for many cancers. Different immunotherapeutic approaches have been utilized in the treatment of haematological malignancies and certain solid tumours, such as advanced melanoma and non-small cell lung cancer, showing impressive results, but unfortunately, the results have been modest in GBM (reviewed in [24,25,26]). GBM is a very heterogeneous disease, and its unique adaptability and resistance to therapies constitute major obstacles for clinical success. The lack of efficacy and resistance to immunotherapy observed are mainly attributed to (i) the highly immunosuppressive tumour microenvironment (TME); (ii) the tumour molecular plasticity and an exceptionally heterogeneous phenotype with intra-tumoral heterogeneity, both across patients and within the same tumour, as well as during tumour progression; (iii) the low mutational burden and low immunogenicity; (iv) the GBM location; indeed, the brain was, for a long time, considered an immune privileged site; and (v) systemic patient immune suppression.

## 4. Tumour Microenvironment (TME) in Glioblastoma

GBM tumour cells originate from astrocytes and cancer stem cells (CSC). Tumour cells interact closely with non-malignant cells, creating a complex and dynamic interplay in the local environment in which the tumour develops and grows. Different types of non-neoplastic cells, both immune and non-immune cells, contribute to the TME structure. Tissue-resident cells are represented by microglia, neurons, vascular endothelial cells, pericytes, fibroblasts and immune cells from both innate and adaptive immunity and can be either resident or infiltrating cells. Myeloid-derived cells and, in particular, tumour-associated macrophages (TAMs) are the most representative immune entity in the TME. The majority of TAMs are infiltrating immune cells that originate from bone marrow myeloid cells, and only a minor population derives from tissue-resident microglia [27,28,29]. Other myeloid-derived cells are represented by infiltrating DCs, monocytes, neutrophils and a less-defined group of cells known as tumour-infiltrating myeloid-derived suppressor cells (MDSCs). TILs represent the adaptive immunity present in the TME and consist of CD8+ cytotoxic T cells (CTLs) and CD4+ helper T cells (Th); of importance, a variable proportion of CD4+ T cells consists of regulatory T cells (Treg).

Neoplastic and non-neoplastic cells create individual structures in distinct regions inside the tumour, named niches, which differ in cell composition and functions (perivascular niche, hypoxic/necrotic core and invasive front), contributing to broad intra-tumour heterogeneity. GBM is also characterized by hypervascularization, which consists of a net of disorganized and abnormal tumour vessels. The abnormal vascularization breaks the properties of the blood–brain barrier (BBB), causing leakage and increasing permeability, which leads to increased immune infiltration, inflammation and oedema in the brain [30]. Concurrently, functional defects of the tumour vessels create regions of hypoxia in the tumour that contribute to reshaping the TME. Overall, GBM TME is an extremely complex ecosystem where tumour cells and other non-malignant cellular entities interact and can play both anti-tumour or pro-tumour functions. They influence each other through the release of cytokines, chemokines, growth factors, metabolites and soluble factors, which remodel the TME continuously and create an immunosuppressive and dynamic milieu that supports tumour growth, adaption, and invasion and contributes to tumour escape and resistance to therapy [31].

Notably, despite its aggressiveness, GBM rarely metastasize outside the brain, suggesting that GBM’s growth is greatly dependent on the unique TME milieu in the brain. It has now become clear that understanding the immune inhibitory phenotype of the TME, its mechanisms and the effects on both immune cells and tumour cells is of critical importance to make significant progress in the treatment of GBM. It is no surprise that targeting the TME has become one of the most pursued strategies for designing novel immunotherapeutic approaches. Strategies tackling the different components of the GBM microenvironment have been tested in preclinical and clinical studies, with the aim of modulating tumour immune suppression and improving the response to immunotherapy. Immunotherapy is based on the use of the patient’s immune cells to fight cancer; therefore, the tumour infiltration of tumour-reactive T lymphocytes, both CTLs and Th cells, is a necessary prerequisite for generating an anti-tumour immune response. Over the years, GBM has been described as an immune desert tumour because of the minimal presence of activated immune cells in tumour specimens. Alternatively, despite the presence of tumour-infiltrating lymphocytes, these immune cells are subjected to several mechanisms of inhibition that lead to different degrees of immune suppression and cell dysfunction, such as anergy, tolerance, senescence and exhaustion [32,33,34]. Effector CD8+ T cells, together with Th1 CD4+ T cells, are major players in the anti-tumour response. In general, the inflamed phenotype “hot tumour” and the presence of CD8+ TILs in the tumour are signs of tumour immunogenicity and have been used as factors to predict a positive response to the treatment in numerous tumours [35,36]. Several studies have shown that TILs in GBM are limited in number, representing less than 0.25% of the total tumour immune cell infiltrate [29,37]. Furthermore, due to a state of chronic inflammation and persistent antigen stimulation, TILs become exhausted and upregulate the expression of multiple inhibitory immune checkpoint receptors, such as PD-1, CTLA-4, TIGIT, LAG-3, TIM-3, CD39 and CD73, while tumour cells and the TME adapt themselves to express the cognate inhibitory ligands [33]. In the CD4+ T-cell compartment, the regulatory CD4+ Tregs seem to play a major role in immune suppression and tumour resistance. Indeed, their number appears to be increased in higher-grade glioma. An increased frequency of Tregs has been correlated with worse prognosis and shorter recurrence-free survival [38,39,40]. Tregs secrete pro-tumorigenic, Th2-polarizing cytokines such as interleukin (IL)-10 and transforming growth factor (TGF)-β, which induce immune suppression and hinder the anti-tumour activity of T cells. GBM, in turn, promotes Treg infiltration and accumulation by secreting soluble factors, including C-C motif chemokines 20 and 22 (CCL20 and CCL22). Earlier studies have suggested a possible positive correlation between the number of TILs and favourable prognosis [41,42]; however, it is important to consider the cell composition of the T-cell infiltrate; for example, in one study, the number of CD4+ T cells was shown to positively correlate with the tumour grade, while the level of CD8+ T cells was inversely correlated [39,40].

### 4.1. Tumour-Associated Macrophages (TAM) and Myeloid-Derived Suppressor Cells (MDSCs)

TAMs represent about 30–50% of the overall cell compartment in GBM, and similarly to Tregs, the number of TAMs has been directly associated with the tumour grade and tumour progression and inversely related to survival [28,43,44,45,46]. TAMs are derived from tissue resident macrophages (MΦs) or from tumour-infiltrating monocytes.

In GBM, the inflammatory milieu, which is characterized by leakage in the BBB and release of several chemoattractant factors, recruits monocytes from the periphery that differentiate into MΦs once infiltrated [47]. MΦs represent a plastic and dynamic cell population in GBM; they can exist in different functional states, but the most predominant phenotype is the so-called anti-inflammatory type M2. The GBM microenvironment produces several factors, monocyte chemoattractant protein-1 (MCP-1/CCL2), colony-stimulating factor-1 (CSF-1), IL-10 and TGF-β, which polarize MΦs toward the pro-tumoral M2 phenotype while reducing the anti-tumoral M1 population. M2 MΦs induce tumour progression by driving tumour proliferation directly or indirectly by dampening T-cell functions through the secretion of cytokines and factors such as TGF-β, IL-10, IL-4, vascular epidermal growth factor (VEGF) and matrix metalloproteases (MMPs). MDSC is a heterogeneous population that arises mainly from monocytes and neutrophils. MDSCs are not present in the brain under non-pathological conditions; however, when cancer and inflammation occur, these cells are recruited into the tumour [48]. Tumour-infiltrating MDSCs mediate potent pro-tumoural action and the immune suppression of T and NK cells through the expression and release of several molecules, such as programmed death-ligand 1(PD-L1), indoleamine 2, 3-dioxygenase (IDO), arginase 1 (ARG1), nitric oxide (NO), reactive oxygen species (ROS), IL-10 and TGF-β [49]. Higher MDSC infiltration has been shown to correlate with poor OS and PFS in patients with solid tumours [50]. Preclinical studies have shown that the different MDSC subsets support GBM growth in a sex-specific manner [51].

### 4.2. GBM Tumour Biology: Molecular Plasticity and Heterogeneity

Intra-tumoural molecular heterogeneity is a barrier to efficient therapy if using mono-targeting therapies and the cause of therapy resistance [52,53]. The targeting of a susceptible malignant clone will provoke the outgrowth of other resistant tumour clones (tumour escape). A longitudinal study showed high heterogeneity between the paired primary tumour and recurrent tumour tissues, where molecularly druggable targets were expressed differentially from diagnosis to tumour progression. Tumour mutational burden (TMB) is the level of genetic mutations in a tumour. Hypermutated tumours generally produce a broader spectrum of cancer mutations, and this increases the chance to have more immunogenic cancer-specific neoantigens and, in turn, a more potent antitumour immune response, which can lead to beneficial therapeutic outcomes. Unfortunately, GBM has a very low tumour mutational load that contributes to its poor immunogenicity [54]; nevertheless, the analysis of several cohorts of recurrent GBM recently showed that very low TMB correlated with the clinical response to immunotherapy [55].

For a long time, the brain was considered a site of immune privilege; indeed, earlier experiments indicated very minimal immune functions in the brain because of a lack of professional antigen-presenting cells (dendritic cells), low levels of MHC classes I and II, the presence of the BBB and the absence of lymphatic vessels [3]. However, this has been revised, as it is now clear that there is communication between the CNS and the immune system, but still, these interactions are unique and more controlled [56].

The BBB plays the first line of defence of the CNS. It blocks the diffusion of large molecules and pathogens into the CNS, also creating a problem for drug delivery when intact. Furthermore, in normal brain conditions, peripheral immune cells (T cells, monocytes and DCs) are excluded. Naïve T cells specific for CNS antigens that enter the CNS are tolerized, and T cells primed in the periphery that reach the CNS are deleted.

In cancer, inflammation and radiotherapy create leakage in the BBB, allowing the entry of immune cells from the periphery. Still, the brain creates a protected environment for GBM, because the compartment is highly sensitive [57].

Overall, the combination of all these unique properties: the immune suppressive TME, the scarce T cell infiltrates, the poor immunogenicity, the molecular heterogeneity and the “immune-privileged site”, contribute to render GBM resistant to immunotherapies.

## 5. Current State and Future of Immunotherapy in GBM

Despite the unfulfilled expectations and the overall failure of different immunotherapeutic interventions in the treatment of GBM, due to a small percentage of GBM patients who experienced clinical benefits and prolonged survival, we still believe that immunotherapy holds the promise to become the standard of care in GBM management. Indeed, recent advancements in immunotherapy strategies, new insights in the biology and mechanisms of GBM and learning from earlier failures will guide the design of the next generation of treatments in GBM.

**Figure 1:**
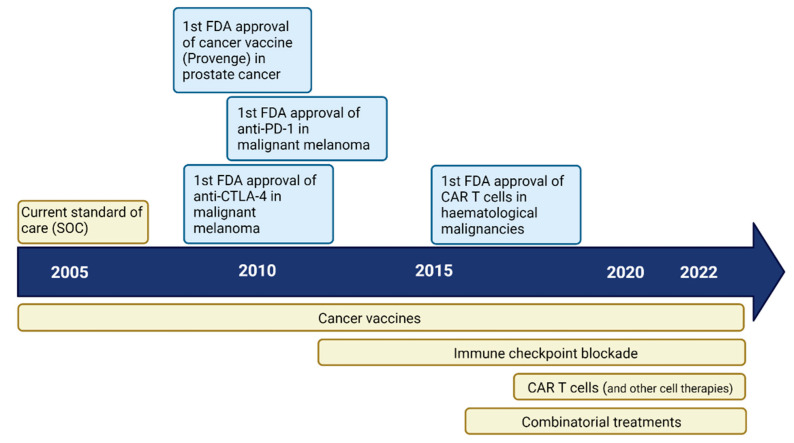
Timeline for immunotherapy development. In blue: immunotherapies approved in other malignancies. In yellow: therapies approved (SOC) or tested in GBM.

Different types of immunotherapy approaches: immune checkpoint blockade (ICB), vaccines, CAR T cells and others, have been utilized to modulate the TME by reducing immune suppression and actively boosting and harnessing the anti-tumour response. Here, we describe the current state-of-the-art immunotherapy and summarize the most relevant clinical trials and if immune correlates have been seen (Table 1, Table 2, Table 3 and Table 4 and Figure 1).

### 5.1. Immune Checkpoint Blockade (ICB)

The results from the randomized clinical trials indicate that glioblastoma are largely resistant to immune checkpoint blockade treatment, except for the rare hypermutated glioblastomas [90,91]. A few ongoing phase I/II or III trials with ICB have still not reached their endpoint. ICB is generally well-tolerated and may be promising in the initial stages of GBM but requires the presence of a pre-existing immune response in the patients to work, as exemplified by the recent approval of ICB in MSI+ glial cell tumours. GBM patient selection in clinical trials of ICB treatment was not based on the presence of pre-existing immune responses or tumour immune cell infiltration. Recent research indicates that T-cell infiltration and markers of dysfunction may be more important than the currently approved biomarkers (PD-L1 expression, MSI and TMB) for accurate prediction of the response to ICB (Table 1). Several large, randomized phase III studies did not meet their clinical endpoint, and no convincing survival benefit was seen after ICB treatment (CheckMate 143 NCT02017717, CheckMate 498 NCT02617589 and CheckMate 548 NCT02667587) (Table 1) [58,59,60]. ICB in neoadjuvant settings have, however, delivered more promising results. Despite no obvious survival benefit, a single-arm phase II study with neoadjuvant anti-PD-1 (Nivolumab) showed a correlation with the enhanced expression of chemokine transcripts in tumour, increased immune cell infiltration and larger TCR clonal diversity among tumour-infiltrating T lymphocytes, indicating increased intra-tumoral immune activity [61].

A recent randomized phase II study (NCT02852655) compared neoadjuvant anti-PD-1 (Pembrolizumab) treatment with continued adjuvant therapy following surgery to adjuvant, post-surgical anti-PD-1 treatment alone [62]. Neoadjuvant anti-PD-1 treatment demonstrated a survival benefit while enhancing both local and systemic antitumour immune responses with the upregulation of T-cell and IFN-γ-related gene expression. Another study of neoadjuvant anti-PD-1 (NCT02337686) has not yet reported on survival but showed scarce CD8+ T-cell and increased MΦ (CD68+) infiltration after treatment [63]. A phase II study treated new unmethylated GBM with anti-PD-L1 (Durvalumab) and radiotherapy (NCT02336165), and the preliminary results reported suggested efficacy [64]. Two other phase II studies recently evaluated anti-PD-L1 (Avelumab) treatment in GBM: one in combination with the VEGFR inhibitor Axitinib (NCT03291314) and one combined with SOC (NCT03047473). Patients with recurrent GBM receiving anti-PD-L1 plus Axitinib failed to meet its primary objective of 6-month PFS of 50%, and unfortunately, no data on immune correlates have been published [65]. The addition of anti-PD-L1 to the SOC post-surgery in primary GBM did not result in any survival benefit but was shown to be safe and with an objective response rate of 23% [66]. A biomarker analysis of pre-treatment biopsies showed that T-cell infiltration was generally low in contrast to microglia/MΦ infiltration, and PD-L1 expression showed no correlation with response to therapy.

A recent study in an orthotopic mouse model of GBM found that mice resistant to ICB had tumour-infiltrating lymphocytes (TIL) with reduced cytotoxic capacity and a more polyclonal TCR repertoire [92]. Tumour-associated macrophages (TAM) were shown to drive this ICB resistance through PD-L1/CD80-mediated CD4+ T-cell suppression and Treg expansion. It remains to be investigated if PD-L1 expression on TAMs is a pre-existing or acquired ICB resistance mechanism and if this could be used as a biomarker to stratify patients.

Clinical data have already confirmed the presence of four immune subtypes of GBM with significant differences in the prognosis and distribution of immune checkpoints, and we believe that identifying these will provide more clinical treatment options with immunotherapy already approved in other cancers (particularly blocking the PD-1/PD-L1 axis) for subgroups of GBM patients [93]. On this note, the analysis of peripheral blood and central and marginal tumour areas in primary and recurrent GBM patients showed an increased presence of blood-derived MΦs in both tumour areas and a higher frequency of infiltrating lymphocytes, with a high level of exhaustion markers in relapsing GBM [94]. A significant inverse correlation between infiltrating T cells and an MDSC subset was also demonstrated, again, showing that the immune cell composition affects the clinical outcome and should be more systematically investigated and linked to therapeutic efficacy.

### 5.2. Therapeutic Vaccines

Cancer vaccines aim at actively triggering and boosting patient’s specific anti-tumour T-cell responses. In the case of GBM, which is a so-defined cold tumour, vaccination might induce tumour-specific T cells that will infiltrate into the tumour. This makes the investigation of antigen-specific immune responses an obvious part of the clinical trials, as the vaccine antigens are normally defined. Unfortunately, the promising findings from the preclinical studies have not translated to the clinic so far; indeed, the clinical outcomes from the phase II/III trials have been modest [95]. Vaccines have proven to induce cellular and humoral anti-tumour responses, and some vaccinated GBM patients have experienced prolonged PFS and OS, but still, the clinical impact on survival needs to be confirmed. Therefore, randomized large size phase III trials should be conducted to confirm the beneficial outcomes from earlier phase I single-arm studies and to prove beyond any doubt the efficacy of vaccination in GBM. On the other hand, vaccination has been effective in a small number of patients bearing the same type of tumour. A deep investigation of the shared characteristics, molecular, biological and immunological, of these long-term survivors might be helpful to understand what conditions make a patient responsive to the vaccine treatment.

In an era where cancer management is moving towards a more targeted and personalized therapy, clinical trials testing the efficacy of distinct immunotherapies should also be more patient-tailored, and clinical trials should be designed to include a less heterogeneous patient population. Ideally, groups of patients that are likely to respond to the therapy should be identified in advance; this could be possible using predictive biomarkers that might be derived from molecular and immunological features, as well as from the clinical history of the patient.

Several vaccine strategies, which diverge in the type of target antigen, number of targeted antigens, platform and mode of delivery, have been evaluated in GBM, but so far, no vaccine has met the primary endpoints in a phase III clinical trial. Here, we have highlighted some of the most relevant clinical studies in relation to the different vaccine modalities and discuss their outcomes and weaknesses (Table 2). Vaccines have been designed to target only one protein, either tumour-specific antigens (TSA) or tumour-associated antigens (TAA). The EGFRvIII mutant arises from a mutation in the receptor of EGFR. EGFRvIII is a tumour-specific antigen expressed on 25–30% of GBM, and for this reason, it has been targeted by several therapies and vaccines. Rindopepimut (CDX-110) is a peptide-based vaccine that targets this mutation. Non-randomized phase II studies of Rindopepimut have shown promising results, with improved progression-free survival (PFS) and overall survival (OS) compared to historical controls [96,97]. These preliminary findings lead to a large, randomized phase III trial (ACT IV, NCT01480479) in newly diagnosed EGFRvIII+ GBM [4]. After tumour resection and standard of care (SOC), patients received either Rindopepimut or a placebo. The trial was suspended because of a lack of effect, and the tumour recurred. Two lessons were learned from this study: (i) even if tumour-specific, targeting of a single antigen is not sufficient to eradicate the tumour if the antigen is expressed heterogeneously and in a fraction of tumour cells, as in the case of EGFRvIII, the mono-targeting strategy might work when the target is expressed on cancer stem cells or if the mutation is essential for cancer cell survival, and (ii) elimination of the antigen-positive tumour cells can induce immune pressure on the tumour, which might lead to the outgrowth of other tumour cell clones, which do not express the vaccine-targeted antigen, and promote tumour escape and progression. In this study, the analysis of the recurrent GBMs showed loss of EGFRvIII in 50% of the cases; however, the disappearance of EGFRvIII+ tumour cells was only due in part to vaccination, since patients in the placebo group also experienced loss of the antigen, indicating that this process was spontaneous. Generally, the recruitment of recurrent GBM patients in vaccine trials has been done on the evaluation of the primary tumours without considering that the relapsed tumour might be molecularly different from the primary tumour; this might explain, in part, the lack or modest effects of the vaccination so far. Other issues of the trial include the use of a strong immune modulator in the control group, which may underscore the efficacy of the vaccine, and lastly, the use of a long peptide that did not induce a strong HLA class I restricted T-cell response, since a humoral response was mainly observed in the trial.

A phase II randomized trial (ReACT, NCT01498328) tested the combination of Rindopepimut and Bevacizumab in recurrent GBM [67]. The results indicated survival benefits and improved OS in the treatment group; however, further validation should be confirmed in a larger size group. EGFRvIII has also been evaluated as a target for CAR therapy in newly diagnosed glioblastoma (NCT02664363), as discussed below [85].

Another recurrent mutation in a subset of GBM patients with improved prognosis is the IDH mutation. The mutation is generally a R132H substitution (IDH1R132H) and represents a promising neoantigen in GBM. Importantly, one study identified immune responses against the IDH1R132H peptide in patients prior to vaccination [98]. Another phase I trial (NCT02454634, NOA-016 trial) showed safety and immunogenicity with the induction of IDH1R132H-specific cell and humoral immune responses [68]. In a later report, the authors communicated that vaccine-induced immune responses were observed in 93.3% and that the 3-year progression-free and death-free rates were 0.63 and 0.84, respectively. Patients that mounted a vaccine-specific response had no tumour progression during this time [99].

Survivin is a protein highly expressed in several tumours, including GBM, while absent in normal differentiated tissues [100]. A peptide-based vaccine targeting survivin (SurVaxM) was evaluated in combination with standard therapy in a phase II trial (NCT 02455557) in newly diagnosed GBM patients and compared to historical controls [69]. The outcomes seemed promising, with a PFS of 11.4 months and OS of 26 months, and randomized studies of SurVaxM are planned in combination with TMZ in naïve GBM and in combination with anti-PD-1 in recurrent GBM.

Aiming to prevent cancer immune escape due to loss of the targeted antigen, multitargeted vaccines have been designed [76]. IMA950 is a peptide-based vaccine that contains 11 GBM-derived non-mutated peptides: nine CD8+ and two CD4+ T-cell epitopes derived from proteins commonly expressed in GBMs [101]. A small phase I clinical trial (NCT01222221) demonstrated vaccine-specific peripheral CD8+ T-cell responses in 90% of patients [70]. In another phase I/II trial (NCT01920191), the IMA950 vaccine was paired with adjuvant poly-ICLC and induced both Th1 CD4+ and CD8+ T-cell responses. CD8+ T-cell responses were specifically directed towards single and multiple peptides [71]. Here, the median overall survival was 19 months, demonstrating the importance of the mixing and co-injection of peptides and adjuvant. Currently, one randomized phase I/II trial testing IMA950 in combination with anti-PD-1 (Pembrolizumab) in relapsing GBM is ongoing (NCT03665545) [72].

The GAPVAC 101 phase I trial (NCT02149225) represents a step further into personalized vaccine therapy. Here, newly diagnosed GBM were vaccinated with two peptide-based vaccines (APVAC), the APVAC1 containing non-mutated peptides derived from known GBM antigens and APVAC2 containing patient-specific neoepitopes [73]. The early results were promising: APVAC1 induced memory immune responses, with CD8+ Tc responses in 12/13 patients and CD4+ Tc responses in 9/13. The APVAC2 neoantigens stimulated mainly Th1 CD4+ Tc responses. The median PFS was 14.2 months, and the median OS was 29 months. A further, larger study in a randomized setting should be run to conclude on the efficacy of the GAPVAC approach.

Personalized peptide-based vaccines targeting neoantigens have also been designed. NeoVax is a patient-tailored multi-epitope vaccine made of 20 long peptides, each containing 3–5 patient-specific neoantigens. In a phase I trial (NCT02287428), a small group of newly diagnosed GBM patients with unmethylated MGMT promoters received NeoVax after surgery and radiotherapy. Patients who required dexamethasone during vaccine priming did not produce a T-cell response, but otherwise, the vaccine stimulated neoantigen-specific immune responses both systemically and in the tumour [74]. Despite the induction of neoantigen-specific responses, the tumour eventually relapsed, and the vaccine responder ultimately died of the disease. The analysis of the relapsed tumour indicated that neoantigen-specific T cells had an exhausted phenotype [74]. General dysfunction of the immune system due to steroid treatment, which is often administered to GBM patients to treat brain oedema, and the exhausted phenotype of the TILs might explain the difficulty in controlling the tumour. The use of immune checkpoint blockade (ICB) in combination with the vaccination could mitigate the exhausted TIL phenotype. The same trial has included a cohort investigating NeoVax in combination with anti-PD-1 (Pembrolizumab), and another trial is testing NeoVax plus anti-CTLA4 (Ipilimumab) or anti-PD-1 (Nivolumab) (NCT03422094). It is relevant to keep in mind that the peptides were selected based on the predicted ability to bind to specific HLA molecules; however, their expression in vivo in tumour cells was never shown. In contrast to the NeoVax study, in the GAPVAC trial [73], the peptides were selected based on peptide elution of the patient’s tumour, reducing the risk that selected neoepitopes are immunogenic but not real, meaning not processed and presented on the surfaces of the tumour cells. Importantly, this study showed that no mutation-derived epitopes were expressed on the tumour cells from the 643 mutations detected in the whole patient group.

A further vaccine approach has used patient-derived dendritic cell (DC) vaccines to present the antigens and prime T cells. DCs can be preloaded with autologous tumour lysate or predetermined tumour-related peptides or can be manipulated genetically to express epitopes from full TAAs or mutated protein. The most advanced DC vaccine, ICT-107, uses autologous DC pulsed with six peptides, two HLA-A1-restricted and four HLA-A2-restricted, which derive from protein expressed and predicted to be abundant in GBM and glioblastoma cancer cells: gp100, MAGE-1, AIM2, HER2, IL13Ra2 and TRP2. A phase I trial showed encouraging results, with patients experiencing immunological responses and reaching a median of 16.9 months PFS and 38.4 months median OS [102]. Patients expressing at least three of the antigens in the vaccine experienced better survival. A randomized phase II trial was started (NCT01280552), and the conclusion was that patients in the HLA-A2 subgroup reached a therapeutic benefit with ICT-107 and elicited an important immunological response [75]. Overall, the primary end point, improved OS, was not reached; however, the efficacy of ICT-107 might be underestimated, since only a fraction of the patients enrolled was HLA-A2+. A phase III trial (NCT02546102) including only HLA-A2+ patients was started and indicated that four of the targeted antigens were associated with better survival; however, the trials were suspended due to a lack of funding.

DCVax-L vaccine consists of autologous DC pulsed with tumour lysate. In a large, randomized phase III trial (NCT00045968), newly diagnosed GBM patients received the standard of care, followed by DCVax-L or a placebo. The authors reported efficacy of the treatment that translated into 23.1 months of median OS vs. 17 months achieved in past studies with SOC [76]. However, interpretation of the data might be misled by several flaws in the trial design; indeed, all relapsing patients, from both groups, were eligible to receive the vaccine, and therefore, about 90% of the patients were finally vaccinated, and it is not clear if the treatment led to extended survival. DCVax-L is also being investigated in combination with anti-PD1 (Nivolumab) in a phase II study (NCT03014804).

The role of Cytomegalovirus (CMV) in GBM is controversial. Several studies have shown the presence of CMV in GBM, whereas CMV proteins were not detected in normal brain tissue [103,104,105]. However, other publications have indicated a lack of CMV protein expression in glioma [106,107]. These inconsistencies might be due to differences in sensitivity detection of the distinct assays. One group in the USA is testing several vaccine trials that target CMV in GBM. One study indicated that patients vaccinated with CMV pp65 mRNA-loaded DCs showed enhanced polyfunctional CMV-specific CD8+ T cells, which seemed to correlate with the OS [108]. These findings need to be corroborated by a larger randomized study. A phase II trial with DC vaccine targeting CMV peptide 65 (ATTAC II; NCT02465268) is ongoing. Other trials with CMV pp65 DC vaccines are currently being tested (NCT02366728 and NCT03688178) [77].

A randomized phase II trial testing a novel vaccine approach (Gliovax, NCT 03395587) is currently ongoing. Gliovax is made of autologous antigens from autologous tumour lysate mixed with lysates from three different allogeneic tumour donors. The vaccine is given with cyclophosphamide to reduce Treg and GM-CSF. In the active group, patients also receive Bevacizumab. Data from the treated cohort are promising in terms of the overall survival [78].

A vaccine approach based on the use of tumour-derived heath-shock proteins has been tested using heat-shock protein complex 96 (HSPPC-96) derived from the patient tumour [109]. A phase I clinical trial (NCT02122822) showed safety of the vaccine and favourable prognosis in patients with improved tumour-specific immune response after vaccination. Tumour-specific immune response after vaccination correlated with enhanced survival [80]. Vaccine-specific immune responses both in the periphery and the tumour were induced. Following these promising results, a phase II trial (NCT03018288) was conducted; here, HSPPC-96 was used in combination with TMZ and Pembrolizumab, but the results are not available [81,82].

Lastly, we report two vaccine trials that have been started at our institution, the Oslo University Hospital. A phase I (NCT00846456) trial was conducted. Patients with unmethylated MGMT-promoter and IDH WT markers received a vaccination with autologous dendritic cells loaded with mRNA-encoding hTERT (telomerase) and survivin, both overexpressed antigens in GBM and tumour stem cell mRNA from autologous tumour spheres. The analysis showed that the vaccine induced a peripheral immune response in all patients. PFS was longer in vaccinated patients compared to matched controls with several patients who were alive at 2 years after diagnosis [79]. Based on these promising results, a randomized phase II/III trial (DEN-STEM, NCT03548571) has been initiated. Interestingly, TCR sequencing data from one of the long-term survivors in the first trial showed large differences in TCR clonality during the disease. TILs from an earlier biopsy were represented by few clones only indicating a very narrow or oligoclonal TCR repertoire, whereas TILs derived from a tumour biopsy at the time of recurrence showed a highly diverse TCR repertoire (unpublished). This difference might be correlated with the evolution of the disease and used to predict the prognosis. It seems reasonable that the over-proliferation of few tumour-specific T-cell clones is a sign of an active immune response trying to eradicate the tumour cells. A polyclonal TCR phenotype is indicative of no or a low response. We are now trying to track some of these TCRs in the different types of samples: PBMC, biopsy and cerebrospinal fluid (CSF), collected during the trial, as well as identifying the antigen specificity of the expanded T-cell clones. We believe that the analysis of effector T cells with TCR repertoire and specific antigens could guide optimal patient stratification to indicate if combination treatment is required. Interestingly, we have isolated several CMV-specific T-cell clones from CSF and tumour tissue in agreement with several studies that consider CMV a potential target for GBM immunotherapy.

Overall, vaccination in GBM seems to hold the potential to induce beneficial clinical outcomes and improve survival and quality of life of the patients. However, as shown from vaccine trials, the results are still unsatisfactory, and several hurdles must be faced. One of the major technical problems has been the design of the trial, size, patient stratification, enrolment and data analysis. Other issues concern the selection of the target; (i) it is clear now that multiple targeting is the strategy to follow, (ii) improvement of the predictor algorithms to select target peptides and test the presence of the epitope in vivo in tumour cells, (iii) choose epitopes that might elicit both CD8+ and CD4+ T-cell responses, (iv) the results indicate that a combination of both shared TAAs and neoantigens should be chosen considering the fact that GBM has a low tumour burden and neoantigens might be few and not optimal. Finally, TILs are exhausted, the TME is highly immunosuppressive, and other treatments like steroids contribute to dampen the systemic immunity.

Despite the new knowledge and implementation of vaccine therapy, the anti-tumour response might be of low magnitude and not able to control and eradicate tumour cells. Large, randomized trials exploiting different types of a combination of vaccines with checkpoint inhibitors and standard therapy should be started, and a standardized immunomonitoring should be done to obtain clue and predictive information to lead to a better management of GBM.

### 5.3. CAR T Cell Therapies

Like cancer vaccine, CAR T-cell therapy exploits specific anti-tumour T-cell responses, but differently from vaccines this strategy is based on the use of autologous or allogeneic T cells that are genetically modified ex vivo to express the CAR. CAR molecules recognize a tumour-specific or -associated surface antigen. CARs are mostly composed of the antigen–recognition domains of an antibody coupled to T-cell receptor intracellular signalling molecules and are thus independent of HLA on the tumour cell surface. The use of CAR T cells in GBM is still at the beginning when compared to other immunotherapies; only a small number of trials have been conducted so far. Results from earlier trials showed the feasibility and safety of the treatment, whereas the clinical efficacy is not yet proven. As for other solid tumours, CAR therapy is facing major challenges, and new strategies are under investigation to improve CAR T-cell tumour infiltration and CAR T-cell persistence, counteract the highly immunosuppressive TME and cope with tumour heterogeneity and antigen loss [110]. Few antigens have been targeted so far in GBM (Table 3).

IL13Ra2 is expressed in about 75% of GBMs, and CAR targeting IL13Ra2 has been designed for GBM treatment. A first-in-human pilot trial was conducted in patients with recurrent GBM who received IL13Ra2 CAR T cells infused several times intra-cranially (NCT00730613) [83]. The trial provided evidence for safety and feasibility of the treatment and despite no achieved survival benefit, the authors observed a transient antitumour response. One patient from a later trial experienced a dramatic clinical and radiographic response, with about 80% tumour shrinkage of all tumour lesions after intra-tumoral and intra-ventricular CAR T-cell infusion (NCT 02208362). The tumour relapsed 7.5 months after the first CAR injection and analysis of the post-treatment biopsy showed the loss of IL13Ra2 [84].

Another pilot phase I trial used a single intravenous dose of EGFRvIII CAR T cells in recurrent GBM (NCT02209376). No clinical responses were observed; however, CAR T cells migrated into the tumour as found in the post-treatment biopsies. Again, the tumour lost expression of the targeted antigen. Furthermore, CAR T-cell infiltration was correlated with a more suppressive TME, as demonstrated by increased numbers of Treg, expression of IDO and PD-L1 [5]. Similarly, another pilot trial (NCT01454596) of third-generation EGFRvIII CAR T cells showed no objective responses nor persistent CAR+ cells were identified [87].

A third CAR against GBM targeted the human epidermal growth factor receptor 2 (HER-2), which is expressed at elevated levels in a subset of GBM [111]. One phase I trial (NCT01109095) utilized virus-specific T cells (CMV, EBV or adenovirus) to express the HER2 CAR. The author reported the safety and presence of CAR T cells for about one year, and they indicated that the disease was stable for a while [86].

Several CAR trials are currently active or have recently been completed with targets: such as IL13Ra2 (NCT02208362), EGFRvIII (NCT02209376, NCT02844062 and NCT03283631) and HER-2 (NCT02442297 and NCT03389230). Alternative CAR targets are also being exploited such as the ephrin type A receptor (EphA) (NCT02575261), PD-L1 (NCT02937844), but we are still awaiting the clinical results.

Emerging targets are under investigation in preclinical and clinical studies. B7-H3 (CD276) is a checkpoint molecule that binds to brain tumours and is a potential target for GBM [112,113,114]. Two early phase trials (NCT04077866, NCT04385173) are currently recruiting recurrent or refractory GBM patients.

An unconventional CAR is the Chlorotoxin (CLTX) peptide-based CAR shown to bind a variety of GBM cell lines and primary GBM that express MMP-2 and to be efficacious in orthotopic xenograft GBM tumour models [115]. The CLTX-CAR is being tested in an ongoing phase I clinical trial (NCT04214392) [88].

One of the emerging CAR targets is CD70, which is implicated in recurrent GBM aggressiveness and maintenance [116]. CD70 CAR T-cell therapy was also shown to significantly reduced GBM growth in a xenograft mouse model [117]. However, as fratricide of CD70+ T cells was seen when the CAR was expressed silencing of CD70 in CAR T cells may be necessary for their production.

Finally, CARs targeting the ganglioside GD2 have shown efficacy against GBM in vitro and in animal models [118,119]. There are ongoing clinical trials in diffuse intrinsic pontine glioma and spinal diffuse midline glioma, but so far, none against GBM (NCT04099797 and NCT04196413).

Preclinical evidence suggests that NK cell-based therapies that are in development for several types of cancers could be interesting for targeting GBM, and there are currently three ongoing clinical trials in GBM, but no clinical results are yet available for these [120,121].

## 6. Combination with Multimodal Therapies

It is clear that GBM as a disease with a very immunosuppressive TME, and most of the immunotherapies have been tested as monotherapies after or in addition to SOC. The current SOC for GBM patients includes surgery, temozolomide chemotherapy, radiotherapy and corticosteroids, which all have additional immunosuppressive effects. There are currently efforts to find synergistic combinations between immunotherapies and the current SOC [122]. Combination therapies targeting the TME may be required to overcome these limitations [123]. Some of the studies testing combinations demonstrate increased clinical efficacies, but this is likely to be further enhanced with improved patient stratification and more personalized therapies (Table 4). Additionally, the combination of ICB with CAR T cells have started to make their way into the clinic in GBM (NCT03726515, NCT04003649 and NCT02873390) [89], but few results are so far available. Furthermore, vaccines have been tested in GBM patients during the past two decades, and some studies have so far tested combinations with ICB in GBM (NCT02529072, NCT03422094), but the results have not been published. Such combinations have recently been shown to significantly improve the clinical response in melanoma patients [124]. The phase III study CheckMate 143 included an arm of patients who received combined Nivolumab and Ipilimumab treatment that was discontinued due to the increased toxicity [6].

Finally, another treatment strategy in GBM that we have not discussed here is the use of oncolytic viruses [125]. Despite the demonstration of clinical benefit in some patients, durable increases in OS have been scarce, but the treatment could possibly be used in combination with immunotherapeutic strategies to increase the efficacy.

Preclinical studies have tested other combinations like anti-PD-1 and anti-CXCR4, which is a chemokine receptor whose overexpression is associated with poor prognosis in GBM [126]. Mice treated with combination therapy demonstrated a significant survival benefit-induced immune memory and decreased tumour infiltration of immunosuppressive myeloid populations. CXCR4-trageting strategies are tested clinically in other cancer types, including myeloma and pancreatic cancer [127,128], and warrant further study in GBM [129,130].

## 7. Discussion

**Figure 2:**
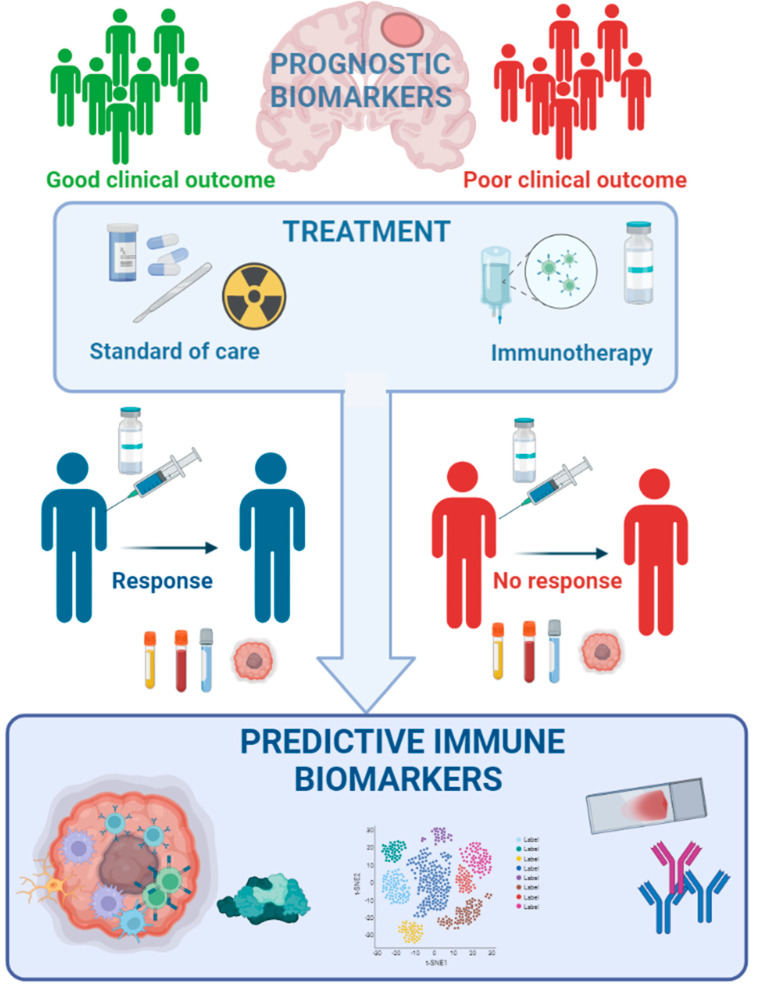
Overview of the prognostic and predictive biomarkers used in GBM patients.

From the different studies discussed with various types of immunotherapy, including ICB, vaccines and CAR T-cell therapy, it is clear that there are immune correlates that associate with a clinical response. In GBM, the assessment of response to immunotherapy relies basically on magnetic resonance imaging (MRI); however, this technology does not allow discrimination between real tumour progression and therapy-induced inflammation (pseudo-progression). Currently, in GBM, the tumour biopsy is used to confirm the disease progression; however, this is a risky intervention considering the location of GBM. Therefore, the validation of novel biomarkers predicting clinical responders is important (Figure 2). For obvious reasons, non-invasive methods of biomarker assessment in peripheral blood would be highly desirable, but at the moment, there are no real blood-based biomarkers for GBM available [131]. Numerous immunological correlates with successful ICB treatment in other cancers could also be relevant for GBM, but these still require further development for use in patient stratification [3,132,133,134,135].

For most of the mentioned therapies, perhaps with the exception of personalized neoantigen-based vaccines, and some CAR T-cell trials, the assessment of target antigen expression prior to enrolment has not been done. This could increase the chance of the response to antigen-specific therapy.

The immune composition of the GBM tumour clearly affects the efficacy of the treatment and immunoprofiling is currently one of the more developed areas of research [136]. Immunosuppression of the patient at the time of diagnosis and in recurrent disease is quite frequent and peripheral lymphocytes, and TILs show an exhausted profile in a large number of patients [33,137].

The standard treatments and the use of steroids contribute even further to the immune inhibition and can dampen the effect of immunotherapeutic interventions by impeding the development of a robust immune response against the tumour. Several studies have pointed to the level of myeloid cell and T-cell infiltration in GBM, the ratios of effector CD8+ T cells: Treg and the level of immune checkpoint molecule expression on intra-tumoural and circulating T cells and myeloid cells. The importance of the PD-1/PD-L1 axis seems relatively established and should be investigated in clinical trials [81,92]. Further characterization of tumour-infiltrating immune cell subsets could lead the identification of novel markers and novel targets. A recent preclinical study identified S100a4 in GBM associated T cells and macrophages as a critical factor promoting immunosuppression and glioma growth [138].

Changes in the TCR repertoire may be another promising biomarker and have been shown to correlate with the treatment efficacy in GBM, but whether increased or decreased, clonality as the preferred outcome depends on the treatment modality. Pre-treatment TCR repertoire evenness was associated with complete response to neoadjuvant chemo-immunotherapy in lung cancer [139]. So far, we have not had sufficient experience with most of these emerging biomarkers to be able to determine thresholds and cut-off values for predicting the response to therapy, and this is likely to vary with the cancer and potential treatment combinations. Well-designed, larger studies are required to ensure this.

Another parameter that will influence the response is the immune competence of the patients that is not tested before enrolment in immunotherapy trials. The ability to mount a peripheral immune response against recall antigens could be easily tested in blood samples and might correlate with a better chance to get an effect from the therapy; however, a peripheral immune response did not directly translate into an effect at the tumour sites [140,141].

The use of validated biomarkers that are currently mainly related to tumour genetics and their landscape, such as TMB, mismatch repair deficiency and PD-L1 expression level, in combination with novel emerging biomarkers linked to the tumour immune infiltration status and TME will help physicians to plan the most appropriate therapeutic interventions, on a case-by-case basis, by identifying which patients are most likely to benefit from the treatment and moving toward a more personalized care. Tumour tissue sampling remains the most precious material for the evaluation of biomarkers, which will help in deciding the type of therapy. Furthermore, the timing of tissue sampling plays a crucial role, because the tumour changes and might progress during treatment. Ideally, sequential biopsies before and during the treatment should be acquired, but in many situations, this is not feasible because of safety reasons, especially in the case of brain tumours.

## 8. Conclusions

GBM remains one of the most treatment-resistant solid tumours with poor prognosis despite the introduction of immunotherapy, which has revolutionized the treatment of many cancers. Neoadjuvant immunotherapies may seem to provide advantages, as they seem to stimulate stronger immune responses than if given concurrently with SOC treatments.

The disease comes with unique challenges due to its location and heterogeneity. This, combined with the profound immunosuppression seen in GBM, will have to be overcome using combination strategies and the enhanced stratification of patients.

For personalized approaches to be effective, predictive biomarkers of response are critical. Numerous clinical studies have observed effects on the immune system correlating with effect of treatment and clinical benefit. Factors including T cell and myeloid tumour infiltration, defined ratios of effector cells versus immunosuppressive cells, expression of immune checkpoint molecules and GBM antigen-specific responses both in circulation and at the tumour site, as well as changes in the TCR repertoire have been correlated with clinical benefit. To become clinically useful these factors need systematic validation in larger patient cohorts to be able to set threshold values for predicting response to treatment and to sample correctly with regards to location and timing. This requires great efforts with respect to clinical trial design but combining prognostic predicators with immune correlates of treatment efficacy will be pivotal for selecting the correct treatment for each patient and improving treatment efficacy in GBM.

## Figures and Tables

**Table 1:** Overview of the immune checkpoint blockade clinical trials in GBM.

Trial Name Clinical Trials.gov Identifier	Phase	Target	Treatment	Indication	Sample Size Recruitment Status	Primary Endpoints	Results	Immunological Response	Comment	Ref.
Immune Checkpoint Blockade (ICB)										
CheckMate 143 NCT02017717	III	PD-1	Nivo −/+ Ipi	rGBM	626 Randomized Active not recruiting Ongoing	OS	No impact	Did not meet primary endpoint		[58]
CheckMate 498 NCT02617589	III	PD-1	Nivo + Rad	MGMT un-methylated nGBM	550 Randomized Completed Ongoing	OS	No impact	Did not meet primary endpoint		[59]
CheckMate 548 NCT02667587	III	PD-1	Nivo + SOC	MGMT methylated nGBM	693 Randomized Active, not recruiting Ongoing	OS	No impact	Did not meet primary endpoint		[60]
MK-3475 NCT02337491	II	PD-1 + VEGF	Pem + Bev	rGBM	80 Randomized Completed Terminated	OS	No impact	Did not meet primary endpoint		
NeoNivo NCT02550249	II	PD-1	Nivo(neoad), surgery, Nivo (ad)	nGBM rGBM requiring surgery	30 Single-arm Completed Terminated	OS	Survival benefit 7.3 months	Increased chemokine transcript expression Immune cell infiltration TCR clonal diversity in tumour.	No obvious clinical benefit	[61]
MK-3475 NCT02852655	I	PD-1	Pem (neoad), surgery, Pem (ad)	rGBM requiring surgery	35 Randomized Completed Terminated	OS	13.7 months vs. 7.5 months	Pre-surgical ICB enables a selective, primary tumour-specific T-cell clonal modulation.	Neoadjuvant ICB enhanced both local and systemic antitumour immune response.	[62]
NCT02337686	II	PD-1	Pem (neoad), surgery, Pem (ad)	rGBM	15 Active, not recruiting Ongoing	PFS6	Unpublished	Rare CD8+ T cells and abundant of CD68+ MΦs in GBM tissue.	Comparison of TIL and PD-L1 scores pre- and post-treatment associated with survival	[63]
Durvalumab NCT02336165	II	PD-L1	Dur + Rad	nGBM un-methylated MGMT	40 Completed Terminated	Safety OS12			First study report of anti-PD-L1 for new GBM	[64]
GliAVax NCT03291314	II	PD-L1 + VEGFR	Ave + Axi	rGBM	52 Completed Terminated	PFS6	No impact		Well-tolerated Did not meet the threshold for activity	[65]
NCT03047473	II	PD-L1	Ave + SOC	nGBM	30 Active, not recruiting Terminated	PFS, OS	Median PFS: 9.7 months Median OS: 15.3 months.	No pre-treatment biomarkers showed any predictive value. No significant treatment effect.	ORR 23.3%	[66]

rGBM, recurrent GBM; nGBM, new GBM; OS, overall survival; PFS, progression-free survival; neoad, neoadjuvant; ad, adjuvant; ORR overall response rate; Nivo, Nivolumab; Ipi, Ipilimumab; Rad, radiation; SOC, standard of care; Pem, Pembrolizumab; Bev, Bevacizumab; Dur, Durvalumab; Ave, Avelumab; Axi, Axitinib.

**Table 2:** Overview of the vaccine and heat-shock protein peptide complex in 96 clinical trials and in GBM.

Trial Name Clinical Trials.gov Identifier	Phase	Target	Treatment	Indication	Sample Size Recruitment Status	Primary Endpoints	Results	Immunological Response	Comment	Ref.
Peptide Vaccine Trials in GBM										
ACT IV (CDX-110) NCT01480479	III	EGFRvIII	Rindo + TMZ	nGBM EGFRvIII+	745 Randomized Completed Terminated	OS	No impact	Increased antigen-specific antibody titres. T-cell response NA.	Loss of EGFRvIII in recurrent tumour	[4]
ReACT NCT01498328	II	EGFRvIII	Rindo + Bev	rGBM EGFRvIII+	70 Randomized Completed Terminated	PFS6	Positive trend Improved OS	Humoral response YES T-cell response NA.	Further validation needed due to small study size.	[67]
NOA-16 NCT02454634	I	IDH1 IDH1R132H mutation	IDH1 vaccine −/+ TMZ	IDH1R132H-mutated, Grade III-IV gliomas	39 CompletedTerminated	Safety Tolerability Immunogenicity	Safe vaccine	Detection of mutation-specific humoral and T-cell responses.	Pseudo progressions after vaccine may indicate intra-tumoural immune reactions	[68]
SurVaxM NCT02455557	II	Survivin	SurvaxM vaccine + TMZ	nGBM	66 Active, not recruiting Ongoing	PFS6	PFS6: 97%, OS12: 94%	Increased survivin-specific IgG titre post-treatment, baseline and CD8+ T-cell responses.	Positive trend. Immunogenicity and minimal toxicity.	[69]
IMA-950 NCT01222221	I	Multi-peptide (IMA-950)	IMA-950 vaccine + SOC	nGBM	45 Completed Terminated	Safety Immunogenicity Response vs. single or multiple tumour-associated peptide	Safe vaccine and immunogenic	Ninety percent of patients showed CD8+ T-cell immune response to at least one TAA, with 50% responding to two or more TAAs.	Steroids did not affect immune responses to vaccine.	[70]
IMA-950 NCT01920191	I/II	Multi-peptide (IMA-950)	IMA-950 vaccine adjuvated with poly-ICLC + SOC	nGBM HLA-A2+	19 Completed Terminated	Safety Tolerability	Safe vaccine and immunogenic	CD8+ T-cell responses to a single or multiple peptides observed in 63.2% and 36.8% respectively. Sustained Th1 CD4+ T-cell responses.	Beneficial effect of adjuvant + vaccines co-injection.	[71]
IMA-950 NCT03665545	I/II	Multi-peptide (IMA-950) + PD-1	IMA-950 /poly-ICLC + anti-PD1 (Pem)	rGBM	24 Randomized Recruiting Ongoing	Incidence of adverse events Safety Tolerability (PFS, OS)		Preliminary results show vaccine-specific CD4 and CD8 T-cell responses in both groups in blood.		[72]
GAPVAC 101 NCT02149225	I	Personalized multiple peptide	APVAC1 + APVAC2 /poly-ICLC + TMZ	nGBM	16 Completed Terminated	Safety Immunological response CD8 specific response	Safe and positive trend for immunological response	Short, non-mutated APVAC1 antigens induced sustained CD8 memory responses. Mutated APVAC2 antigens induced predominantly CD4 Th1 type responses.	Median PFS and OS: 14.2 and 29 months from diagnosis, respectively.	[73]
NeoVax NCT02287428	I	Personalized neoantigen peptide −/+ PD-1	NeoVax + TMZ −/+ Pem	MGMT un-methylated nGBM	56 Recruiting Ongoing	Feasibility and safety	Pending	In no dexamethasone patients circulating polyfunctional neoantigen-specific CD4+and CD8+T-cell responses enriched in a memory phenotype. Increased number of TILs.	Neoantigen-specific T cells from blood can migrate into tumour.	[74]
Dendritic Cell (DC) Vaccine Trials in GBM										
ICT-107 NCT01280552	II	Autologous DCs pulsed with peptides targeting GBM tumour/stem cell-associated antigens	ICT-107 DC vacc + TMZ	nGBM HLA-A1+ and/or HLA-A2+	278 Randomized Completed Terminated	OS OS in HLA-A2	No difference in OS. PFS significantly improved	Robust systemic response HLA-A2 subgroup showed increased ICT-107 activity clinically and immunologically	HLA-A2 primary tumour antigen expression was higher than for HLA-A1 HLA-A2 patients had higher immune response and meaningful therapeutic benefit whereas only HLA-A1 MGMT methylated patients had an OS benefit.	[75]
ICT-107 NCT02546102	III	Autologous DCs pulsed with peptides targeting GBM tumour/stem cell-associated antigens	ICT-107 DC vacc + TMZ	nGBM HLA-A2+	14 Randomized Suspended (lack of funding)	OS				
DCVax-L NCT00045968	III	Autologous DCs pulsed with tumour lysate	DCVax-L + SOC	nGBM	348 Randomized Unknown Completed	PFS	23.1 months median OS vs. 17 months	Increased frequency of CD4+ T cells	Due to the crossover design, nearly 90% of the population received DCVax-L at some point in the trial.	[76]
DCVax-L NCT03014804	II	Autologous DCs pulsed with tumour lysate −/+ PD-1	DCVax-L + SOC −/+ Nivo	rGBM	0 Withdrawn	Safety and tolerability	None		Withdrawn (Final contract negotiations)	
ATTAC II NCT02465268	II	CMV pp65 autologous DCs	pp65 DC vaccine	nGBM	175 Randomized Recruiting Ongoing	OS				[77]
ELEVATE NCT02366728	II	CMV pp65-LAMP mRNA, autologous DCs	Benefit of tetanus-diphtheria (Td) toxoid pre-conditioning on DC migration and evaluation of synergy among vaccination	GBM	64 Randomized Completed Terminated	OS	Not yet available		Confirmed that pre-conditioning with (Td) toxoid significantly increased DC migration to the lymph nodes.	[77]
DERIVe NCT03688178	II	CMV pp65-LAMP mRNA, autologous DCs	Benefit of Td toxoid pre-conditioning on DC migration and evaluation of synergy among vaccination	GBM	112 Randomized Recruiting Ongoing	Safety OS				[77]
GLIOVAX NCT03395587	II	Tumour lysate-loaded mature DCs	DC vaccine + SOC	GBM	136 Randomized Recruiting Ongoing	OS	No impact		Encouraging, but cannot provide robust evidence of clinical efficacy because of non- controlled studies or low patient numbers.	[78]
NCT00846456	I/II	DCs with mRNA from tumour stem cells + hTert/Survivin mRNA	DC vaccine with mRNA from tumour stem cells + hTert/Survivin mRNA	GBM	20 Completed Terminated	Safety, Immunological response	PFS longer compared to matched control patients	Peripheral vaccine-induced immune response	Several patients alive at 2 years after diagnosis.	[79]
DEN-STEM NCT03548571	II/III	DCs with mRNA from tumour stem cells + hTert/Survivin mRNA	DC vaccine with mRNA from tumour stem cells + hTERT/Survivin mRNA	GBM	60 randomized Active	PFS	Not yet available			
Heat Shock Protein Complex Trial in GBM										
Heat Shock Protein gp96 NCT02122822	I	HSP gp96-peptide complex from patient’s tumour cells	HSPgp96 vaccination + SOC	nGBM	20 Completed Terminated	Safety and effectiveness	Safe and effective	Tumour-specific immune response was significantly increased after vaccination	Tumour-specific immune response after vaccination, instead of which before vaccination, correlated with good survival in vaccinated patients.	[80]
Heat Shock Protein gp96 NCT03018288	II	HSP gp96-peptide complex from patient’s tumour cells + PD-1	HSP gp96 vaccination + SOC −/+ Pem	nGBM	90 Randomized Active, not recruiting Ongoing	1 year OS	Pending			[81,82]

rGBM, recurrent GBM; nGBM, new GBM; OS, overall survival; PFS, progression-free survival; TMZ, Temozolomide; Nivo, Nivolumab; Rad, radiation; SOC, standard of care; Pem, Pembrolizumab; Bev, Bevacizumab; Td, tetanus–diphtheria toxoid.

**Table 3:** Overview of the CAR clinical trials in GBM.

Trial Name Clinical Trials.gov Identifier	Phase	Target	Treatment	Indication	Sample Size Recruitment Status	Primary Endpoints	Results	Immunological Response	Comment	Ref.
CAR T cell Trials in GBM										
IL13Ra2 NCT00730613	I	IL13Ra2	IL13Ra2 CAR intracranial CD3z 1st generation CAR	rGBM	3 Completed Terminated	Safety and feasibility	Safe and feasible No survival benefit	Evidence for transient anti-glioma responses was observed in 2 of the patients. Reduced IL13Rα2 expression within the tumour following treatment.	First-in-human pilot	[83]
IL13Ra2 NCT02208362	I	IL13Ra2	IL13Ra2 CAR 4-1BB-CD3z 2nd generation Intracavitary and intraventricular infusions	rGBM	82 Active, not recruiting Ongoing	Safety and feasibility	Pending	One patient had dramatic clinical response sustained for 7.5 months. Reduction in size of all intracranial and spinal tumours.		[84]
ExCeL NCT02664363	I	EGFRvIII	EGFRvIII CAR + TMZ^DI^ (dose-intensified)	nGBM	3 Terminated (Study funding ended) Terminated	Max tolerated dose Safety	Safe Feasible	TMZ^DI^ pre-treatment prompted dramatic CAR proliferation and enhanced persistence in circulation.		[85]
EGFRvIII NCT02209376	I	EGFRvIII	EGFRvIII CAR 4-1BB-CD3z 2nd generation CAR	rGBM	11 Terminated by the sponsor	Safety and feasibility	No clinical response	Detectable transient expansion of CAR T EGFRvIII cells in peripheral blood. CAR T EGFRvIII migrated into the tumour. Increased expression of inhibitory molecules and infiltration by regulatory T cells after CAR T EGFRvIII infusion.		[5]
HER2 NCT01109095	I	HER2 virus specific	Virus-specific T cells expressing HER2 CAR 2nd generation	rGBM	16 Completed Terminated prematurely	Safety and feasibility	Median OS of 11.1 months after T-cell infusion and 24.5 months after diagnosis.		Three patients alive with no disease progression at last follow-up.	[86]
EGFRvIII NCT01454596	I/II	EGFRvIII	EGFRvIII CAR CD28-4-1BB-CD3z 3rd generation	rGBM	18 Completed Terminated	Safety, Feasibility, PFS6	no OR			[87]
EGFRvIII NCT02844062	I	EGFRvIII	EGFRvIII CAR	rGBM	20 Unknown Terminated	Safety, Feasibility				
EGFRvIII NCT03283631	I	EGFRvIII	EGFRvIII CAR	GBM	24 Terminated Terminated	Max tolerated dose				
HER2 NCT02442297	I	HER2	HER2 CAR 2nd generation CAR T cells	GBM	28 Recruiting Ongoing	Safety				
HER2 NCT03389230	I	HER2	HER2 CAR 4-1BB 2nd generation CAR T cells	GBM	42 Recruiting Ongoing	Safety				
EphA2 NCT02575261	I/II	EphA2	EphA2 autologous CAR T cells	GBM EphA2+	0 Withdrawn	Safety, effectiveness				
Anti-PD-L1 CSR T cells NCT02937844	I	Anti-PD-L1 chimeric switch receptor	Chimeric switch receptor with PD-1 extracellular domain fused to the costimulatory molecule CD28.	rGBM	20 Unknown Terminated	Safety, Efficacy				
B7-H3 CAR T cells NCT04077866	I/II	B7-H3	B7-H3 autologous CAR T cells + TMZ	rGBM	40 Randomized Recruiting Ongoing	Safety, Efficacy, OS				
B7-H3 NCT04385173	I	B7-H3	B7-H3 autologous CAR T cells + TMZ	rGBM	12 Recruiting Ongoing	Safety, Feasibility, OS, PFS				
Chlorotoxin NCT04214392	I	Chlorotoxin tumour-targeting domain	Chlorotoxin-CD28-CD3zeta 2nd generation CAR	rGBM	36 Recruiting Ongoing	Toxicity, Safety			Strong CLTX binding to tumour cells was observed in of the majority of primary GBM lines.	[88]

rGBM, recurrent GBM; nGBM, new GBM; OS, overall survival; PFS, progression-free survival; TMZ Temozolomide.

**Table 4:** Combinatorial clinical trials in GBM.

Trial Name Clinical Trials.gov Identifier	Phase	Target	Treatment	Indication	Sample Size Recruitment Status	Primary Endpoints	Results	Immunological Response	Comment	Ref.
Combinatorial Trials in GBM										
NCT03726515	I	EGFRvIII + PD-1	EGFRvIII CAR-T + Pem	EGFRvIII+, MGMT unmethylated nGBM	7 Completed Terminated	Safety				[89]
NCT04003649	I	IL13Ra2 −/+ PD-1 −/+ CTLA-4	IL13Ra2-CAR T cells +/− Nivo and Ipi	rGBM	60 Randomized Recruiting	Adverse events, Toxicity, Feasibility, OS				
NCT02873390	I	PD-1/EGFR	PD-1 Antibody expressing CAR-T cells for EGFR+ advanced solid tumour	Advanced malignancies incl. GBM	20	OR, PFS, OS				
AVERT NCT02529072	I	PD-1	Nivo with DC vaccines for recurrent brain tumours	GBM	6 Randomized Completed	Safety				
NeoVax NCT03422094	I	Personalized neoantigen peptide vaccine + PD-1 −/+ CTLA-4	NeoVax+ TMZ+ Ipi −/+ Nivo	MGMT unmethylated nGBM	3 Terminated	Safety, Feasibility, Immunogenicity				

rGBM, recurrent GBM; nGBM, new GBM; OS, overall survival; PFS, progression-free survival; Nivo, Nivolumab; Ipi, Ipilimumab; Rad, radiation; SOC, standard of care; Pem, Pembrolizumab; TMZ Temozolomide.
